# Quercetin Prevents Diastolic Dysfunction Induced by a High-Cholesterol Diet: Role of Oxidative Stress and Bioenergetics in Hyperglycemic Rats

**DOI:** 10.1155/2018/7239123

**Published:** 2018-01-11

**Authors:** Rodrigo L. Castillo, Emilio A. Herrera, Alejandro Gonzalez-Candia, Marjorie Reyes-Farias, Nicole de la Jara, Juan Pedro Peña, Catalina Carrasco-Pozo

**Affiliations:** ^1^Pathophysiology Program, ICBM, Faculty of Medicine, University of Chile, Av. Salvador 486, Providencia, 7500922 Santiago, Chile; ^2^International Center for Andean Studies, Universidad de Chile, Putre, Chile; ^3^Department of Nutrition, Faculty of Medicine, University of Chile, Santiago, Chile; ^4^Universidad de Viña del Mar, Región de Valparaíso, Chile; ^5^Centro de Simulación Clínica, Universidad Iberoamericana, Santiago, Chile; ^6^Servicios Médicos Veterinarios de Especialidad-VETCO, Santiago, Chile; ^7^Discovery Biology, Griffith Institute for Drug Discovery, Griffith University, Nathan, QLD 4111, Australia

## Abstract

Alterations in cardiac energy metabolism play a key role in the pathogenesis of diabetic cardiomyopathy. Hypercholesterolemia associated with bioenergetic impairment and oxidative stress has not been well characterized in the cardiac function under glycemic control deficiency conditions. This work aimed to determine the cardioprotective effects of *quercetin* (QUE) against the damage induced by a high-cholesterol (HC) diet in hyperglycemic rats, addressing intracellular antioxidant mechanisms and bioenergetics. Quercetin reduced HC-induced alterations in the lipid profile and glycemia in rats. In addition, QUE attenuated cardiac diastolic dysfunction (increased E:A ratio), prevented cardiac cholesterol accumulation, and reduced the increase in HC-induced myocyte density. Moreover, QUE reduced HC-induced oxidative stress by preventing the decrease in GSH/GSSG ratio, Nrf2 nuclear translocation, HO-1 expression, and antioxidant enzymatic activity. Quercetin also counteracted HC-induced bioenergetic impairment, preventing a reduction in ATP levels and alterations in PGC-1*α*, UCP2, and PPAR*γ* expression. In conclusion, the mechanisms that support the cardioprotective effect of QUE in rats with HC might be mediated by the upregulation of antioxidant mechanisms and improved bioenergetics on the heart. Targeting bioenergetics with QUE can be used as a pharmacological approach to modulate structural and functional changes of the heart under hypercholesterolemic and hyperglycemic conditions.

## 1. Introduction

Hypercholesterolemia is a major risk factor for developing cardiovascular diseases. It further induces oxidative stress, resulting in increased lipid peroxidation in multiple organs [1]. Previous studies have also indicated that hypercholesterolemia is causally linked to a significant increase in reactive oxygen species (ROS) with concomitant lower antioxidant capacity in cardiac tissue [2, 3]. In addition, high cholesterol (HC) levels may activate endothelial cells and lead to increased production of ROS [4, 5]. This mechanism induces vascular function impairment, cell proliferation, cell death, and cardiac remodeling [6, 7]. Indeed, a HC diet leads to oxidative stress, spontaneous arterial vasoconstriction, and systemic hypertension [8] as well as Western diet-induced biventricular cardiomyocyte hypertrophy, increased stiffness, and impaired relaxation in rats [9]. However, the effects of a high-cholesterol diet on bioenergetics and oxidative stress on the impairment of the cardiac function are only partially known and no treatment has shown compelling effectiveness.

Over the last decade, a growing interest has been focused on dietary flavonoids with antioxidant properties such as radical scavenging, metal chelating, and antioxidant enzyme modulation [10]. More recently, it has been shown that flavonoids can also improve intracellular bioenergetics [11, 12]. These compounds could exert cardioprotective effects due to their ability to attenuate oxidative stress, among others. Interestingly, other key mechanisms to preserve cardiovascular function, such as energy supply and turnover, have not been characterized in response to these compounds. Quercetin (QUE) is the most abundant and common flavonoid in the human diet, emphasizing its high antioxidant activity [13]. Quercetin has been shown to protect against mitochondrial dysfunction induced by a HC diet in the pancreas, preserving bioenergetics of pancreatic *β*-cells [12]. The cardioprotective effects of QUE supplementation have been studied in paradigms of myocardial and vascular injury mediated by oxidative stress [14, 15]; however, the molecular mechanisms involved in the functional protective response have not been fully characterized.

We hypothesized that QUE has cardiac protective effects exerted by the upregulation of intracellular antioxidant mechanisms and by the improvement in bioenergetics. Therefore, the aim of this study was to determine the cardiac function, antioxidant capacity, and bioenergetic molecular mechanisms supporting the cardiac effects of QUE supplementation in rats fed with a high-cholesterol diet.

## 2. Materials and Methods

### 2.1. Animals and Diets

The study protocol was approved by the Animal Ethics Committee of the Faculty of Medicine of the University of Chile (CBA#0586 FMUCH), and all procedures were performed in compliance with the Guidelines for Care and Use of Laboratory Animals.

Forty male Wistar rats (90–110 g, 5-6 weeks old) from the Faculty of Medicine were housed in a 12 h light/dark schedule at room temperature with water ad libitum and were randomly distributed into 5 groups. These groups consisted in (1) standard diet fed (C, *n* = 8; AIN-76A/Clinton-Cybulsky Cholesterol Series #1–107); (2) standard diet supplemented with quercetin (CQ, *n* = 8, 0.5% *w/w*); (3) high-cholesterol diet (HC, *n* = 8, 1.25% cholesterol *w/w*, AIN-76A/Clinton-Cybulsky Cholesterol Series #3–107); and (4) HC diet supplemented with either quercetin (HCQ, *n* = 8, 0.5% w/w) or (5) ezetimibe (HCE, *n* = 8, 0.001% *w/w*) for 4 weeks. Ezetimibe blocks Niemann-Pick C1 Like 1 (NPC1L1) protein in the small intestine, a transporter that mediates cholesterol absorption [16]. The doses of quercetin and ezetimibe used in this study are based in a previous study developed by our group [12]. Quercetin was obtained from Sigma-Aldrich (catalogue number 337951, Germany), and ezetimibe was from MSD Chile (Ezetrol®, Chile).

### 2.2. Echocardiographic Analysis

After 4 weeks of treatment, ultrasound and Doppler examinations were performed to the animals under ketamine:xylazine anaesthesia (80 mg/kg:10 mg/kg, i.p.) using a Sonosite 180 plus echocardiograph equipped with an electronic 10 MHz linear-array transducer. During the echocardiographic examination, we determined the wall thickness (AWT), left and right ventricular end-systolic cavity (L-R, VESC), and left and right end diastolic cavity (L-R, VEDC). Ejection fraction (EF, %) and fractional shortening (FS, %) were calculated according to the formulas EF = [(LVEDV − LVESV)/LVEDV] × 100(%) and FS = [(LVEDC − LVESC)/LVEDC] × 100(%) [17]. Further, aortic maximal and mean velocity (Vmax and Vmean) and mean peak gradient (mPG) were assessed by power Doppler.

Transmitral inflow Doppler obtained in an apical 4-chamber view or LV long-axis view was use for the evaluation of LV diastolic function [18]. The Doppler indexes include the ratio of peak velocity of early to late filling of mitral inflow (E/A) and the isovolumetric relaxation time (IVRT).

### 2.3. Sample Collection and Determinations

#### 2.3.1. Basal Blood Glucose

By the end of the 4 weeks of treatment, tail blood glucose levels were determined during the morning in 12 h-fasted animals using an Accu-chek glucometer (Roche, Mannheim, Germany).

Twenty-four hours after, we collected central venous blood and heart from animals euthanised under ketamine:xylazine anaesthesia (100 mg/kg:10 mg/kg, i.p.) and posterior exsanguination.

#### 2.3.2. Plasma Cholesterol

Cholesterol was determined using a colorimetric enzymatic kit (Colestat, Wiener Lab, Argentina). Further, lipid profile (LDL, VLDL, and HDL) was performed by Laboratorio Clinico de Medicina Nuclear (Santiago, Chile).

#### 2.3.3. Heart Collection

The hearts were removed and weighed. Half of the cardiac tissue was stored in 4% paraformaldehyde and embedded in paraffin for histological analyses, and the other half was frozen and stored at −80°C for biochemical and molecular biology analyses.

### 2.4. Cardiac Histology

Heart tissue was washed in PBS immersed-fixed with 4% PFA for 24 h at 4°C. Fixed samples were embedded in paraffin and cut in 4 *μ*m slides. Hematoxilin-Eosin and Van Gieson staining were performed for myocardial density in images captured at 40x and 400x with a digital camera coupled to a microscope (Olympus BX-41). The analysis of the microphotographs was performed with the software Image Pro-Plus 6.2 (Media Cybernetics Inc., Rockville, MD, USA). Briefly, the complete cardiac analysis consisted in the determination of the luminal area of the left and right ventricles and the thickness of the septum and both ventricular free walls. In addition, for each ventricle wall and septum, we calculated the myocardial cellular density as the number of nuclei/area.

### 2.5. Oxidative Stress Markers

The intracellular redox status in cardiac tissue was assessed by a fluorometric method in order to measure the oxidized glutathione (GSSG) and reduced glutathione (GSH) and to determine the GSH/GSSG ratio [19]. The inter- and intra-assay CVs for GSH and GSSG were 3.1% and 4.2%; and 2.7% and 3.5%, respectively.

Lipid peroxidation was assessed by the thiobarbituric acid reaction at pH 3.5, followed by solvent extraction with a mixture of n-butanol/pyridine (15 : 1, *v/v*) [20]. Tetramethoxypropane was used as the external standard, and the levels of lipid peroxides were detected spectrophotometrically at 532 nm and were expressed as mmol TBARS/mg protein. The interassay and intra-assay CVs for TBARS were 10.5% and 4.8%, respectively.

Plasma 8-isoprostane concentration (pmol/mL) was determined using an ELISA kit (Cayman, Ann Arbor, MI, USA) [Milne, 2005]. The inter- and intra-assay CVs were 9.5% and 10.7%, respectively.

### 2.6. Antioxidant Defenses

Heart lysates were homogenized in 0.25 M sucrose for the determination of Cu-Zn superoxide dismutase (SOD) activity or in 1.15% KCl 10 mM Tris/HCl buffer (pH 7.4) for catalase (CAT) and glutathione peroxidase (GSH-Px) activities. The Cu-Zn SOD assay is based on the SOD-mediated increase in the rate of autooxidation of catechols in an aqueous alkaline solution in order to yield a chromophore with a maximum absorbance at 525 nm [21]. One Cu-Zn SOD unit is defined as the activity that doubles the autooxidation background, and the results are expressed as units/mg of protein. CAT activity was assayed by the breakdown kinetic of peroxide of hydrogen (H_2_O_2_) at 240 nm and was express as the first-order reaction rate constant (k)/mg of protein [22]. Soluble GSH-Px activity was measured spectrophotometrically in a cytosolic fraction by the reduction of glutathione disulfide coupled to NADPH oxidation by glutathione reductase [23]. One GSH-Px unit is defined as the activity that oxidizes 1 *μ*mol of NADPH/min and is expressed as units/mg of protein.

To evaluate Nrf2 translocation, nuclei were extracted from cardiac tissue as previously described [24] and an Nrf2 transcription factor assay kit was used to assess Nrf2 DNA binding activities (Cayman, MI, USA).

### 2.7. Transcript Measurements

The mRNA levels of *HO-1*, peroxisome proliferator-activated receptor gamma coactivator 1-alpha (*PGC-1α*), and uncoupling protein 2 (*UCP2*) were evaluated by qPCR [25]. The expression of each gene is relative to the cycle thresholds of two housekeeping genes, *Actb* and *GADPH*. Primer sequences are provided in Supplementary Table 1.

### 2.8. Statistical Analysis

Data were analysed by one-way or two-way ANOVA, followed by Tukey's multiple comparison test using GraphPad Prism 6 (La Jolla, CA, USA). Unless indicated otherwise, the biochemical and molecular biology experiments were performed in triplicate or quadruplicate. Values are expressed as mean ± SEM.

## 3. Results

### 3.1. Quercetin Ameliorates Cholesterol-Induced Alterations in the Lipid Profile

**Figure 1:**
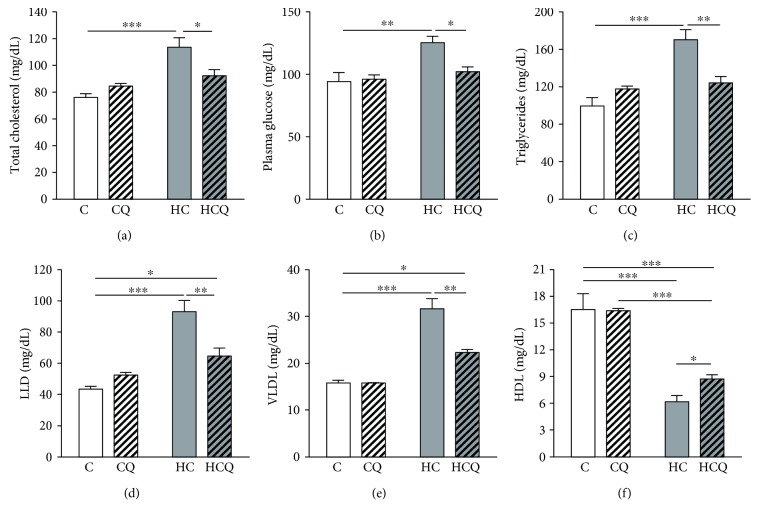
Quercetin protects against the alteration on plasma cholesterol and glycemia levels and lipid profile induced by high-cholesterol diet. Plasma levels of (a) total cholesterol, (b) glucose, (c) triglycerides, (d) LDL, (e) VLDL, and (f)HDL from 12 h fasted rats, fed for 4 weeks with control diet (C), control diet containing 0.5% quercetin (CQ), high-cholesterol diet (HC), or high-cholesterol diet containing 0.5% quercetin (HCQ). Values are expressed as mean ± SEM. *N* = 6–8 rats/group. Two-way ANOVA test and Bonferroni posttest. Statistical differences: ^∗^ *p* < 0.05, ^∗∗^ *p* < 0.01, and ^∗∗∗^ *p* < 0.001.

Four weeks of high-cholesterol (HC) feeding in rats resulted in a 74% and 36% increase in total plasma cholesterol and glucose levels, respectively (Figures 1(a) and 1(b)). HC diet increased the triglycerides, LDL, and VLDL plasma levels by 70%, 114%, and 100%, respectively, and reduced the HDL plasma levels by 63% (Figures 1(c), 1(d), 1(e), and 1(f)). Quercetin fully prevented the HC-induced increase in the plasma levels of total cholesterol, glucose, and triglycerides (Figures 1(a), 1(b), and 1(c)). In addition, QUE only prevented the increase in LDL by 57% and VLDL by 59% (Figures 1(d) and 1(e)). Furthermore, QUE prevented the decrease in HDL by 25% (Figure 1(f)). Quercetin had no effect on cholesterol levels and lipid profile when supplemented the control diet. Conversely, ezetimibe totally prevented the increase in the plasma levels of total cholesterol (Supplementary Figure 1A) and glucose (data not shown) induced by HC diet.

### 3.2. Quercetin Prevented Cholesterol-Induced Cardiac Dysfunction

**Figure 2:**
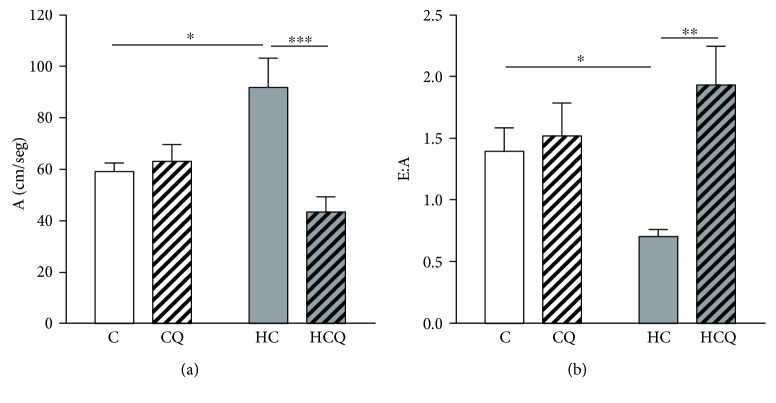
Quercetin protects against cardiac diastolic dysfunction induced by high-cholesterol diet. Ultrasound and Doppler imaging showing (a) A wave (atrial filling velocity) expressed as cm/seg and (b) E:A, expressed as the ratio of peak velocity of early to late filling of mitral inflow, in rats fed for 4 weeks with control diet (C), control diet containing 0.5% quercetin diet (CQ), high-cholesterol diet (HC), and high-cholesterol diet containing 0.5% quercetin (HCQ). Values are expressed as mean ± SEM. *N* = 6 rats/group. Two-way ANOVA test and Bonferroni posttest. Statistical differences: ^∗^ *p* < 0.05, ^∗∗^ *p* < 0.01, and ^∗∗∗^ *p* < 0.001.

The HC diet increased the A wave by 55% and decreased the E:A ratio by 50% (Figures 2(a) and 2(b)). Quercetin totally prevented the HC-induced alterations on cardiac diastolic function (Figures 2(a) and 2(b)). In contrast, cardiac systolic (EF, % and SF, %) and structural (left and right) ventricular parameters were similar between groups (Table 1).

### 3.3. Quercetin Prevented Cholesterol-Induced Composition and Morphological Alterations in the Heart

**Figure 3:**
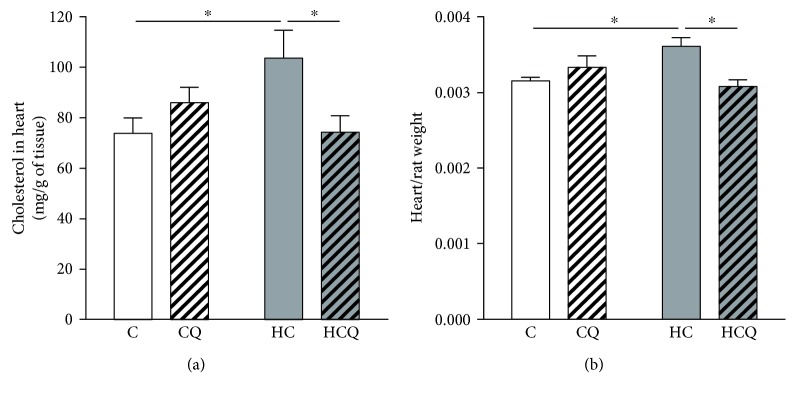
Quercetin protects against composition and morphological alterations induced by high-cholesterol diet. (a) The content of cholesterol and (b) weight of the heart were assessed in rats fed for 4 weeks with control diet (C), control diet containing 0.5% quercetin (CQ), high-cholesterol diet (HC), or high-cholesterol diet containing 0.5% quercetin (HCQ). Values are expressed as mean ± SEM. *N* = 6–8 rats/group. Two-way ANOVA test and Bonferroni posttest. Statistical differences: ^∗^ *p* < 0.05.

The HC diet increased the accumulation of cholesterol in cardiac tissue by 41% and the weight of the heart by 15% (Figures 3(a) and 3(b)). However, quercetin prevented the HC-induced increase in cardiac cholesterol and weight of the heart (Figures 3(a) and 3(b)). Quercetin had no effect on the accumulation of cholesterol in cardiac tissue and on the weight of the heart when supplemented the control diet. Interestingly, ezetimibe also prevented the increase in cardiac cholesterol and weight of the heart induced by HC diet (Supplementary Figures 1B and 1C).

**Figure 4:**
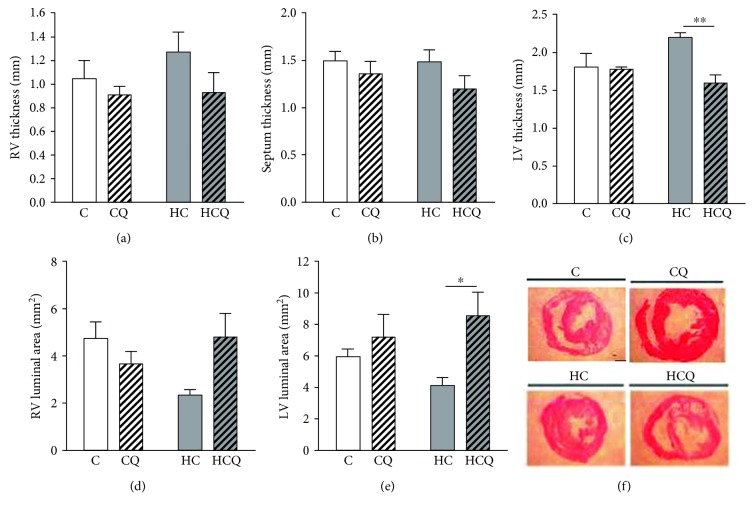
Quercetin protects against cardiac wall remodeling induced by high-cholesterol diet. (a) Right ventricle, (b) septum, and (c) left ventricle thicknesses; (d) right ventricle and (e) left ventricle luminal areas. (f) shows representative pictures of each group (millimetric bar: 2 mm). Heart dimensions were assessed in rats fed for 4 weeks with control diet (C), control diet containing 0.5% quercetin (CQ), high-cholesterol diet (HC), or high-cholesterol diet containing 0.5% quercetin (HCQ). Values are expressed as mean ± SEM. *N* = 6–8 rats/group. Two-way ANOVA test and Bonferroni posttest. Statistical differences: ^∗^ *p* < 0.5 and ^∗∗^ *p* < 0.01.

The right ventricle wall and septum thickness were similar between the four experimental groups. However, the left ventricle wall thickness was increased in HC, an effect that was reverted by quercetin treatment (Figures 4(a), 4(b), and 4(c)). In addition, the right ventricle luminal area was similar between groups, although there was a clear tendency to decrease in HC group that was not seen in the HCQ animals (Figure 4(d)). By contrast, the left ventricle luminal area was significantly decreased in HC animals, and this effect was fully reverted by quercetin (Figure 4(e)).

**Figure 5:**
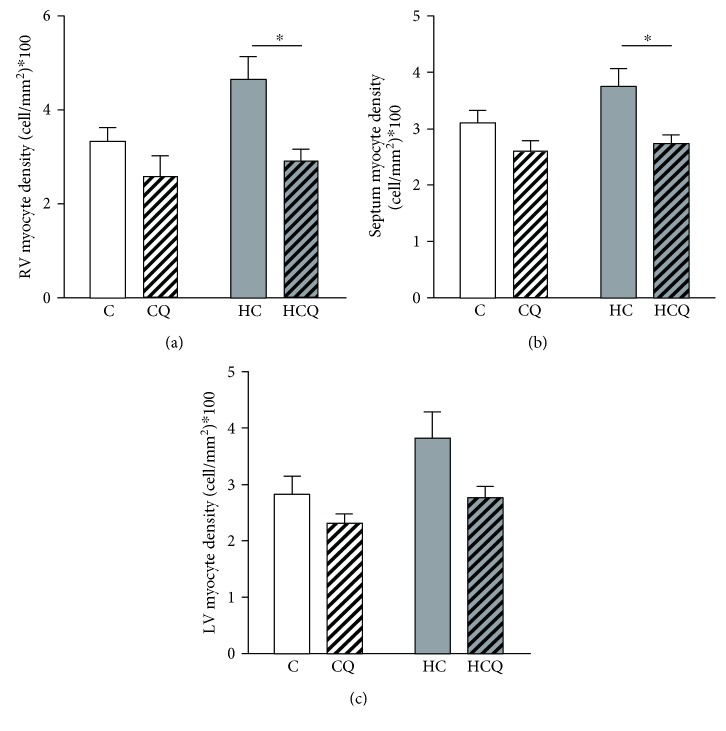
Quercetin protects against increased myocyte density induced by high-cholesterol diet. (a) Right ventricle, (b) septum, and (c) left ventricle myocyte densities, assessed in rats fed for 4 weeks with control diet (C), control diet containing 0.5% quercetin (CQ), high-cholesterol diet (HC), or high-cholesterol diet containing 0.5% quercetin (HCQ). Values are expressed as mean ± SEM. *N* = 6–8 rats/group. Two-way ANOVA test and Bonferroni posttest. Statistical differences: ^∗^ *p* < 0.05.

Myocite density tends to increase in the right ventricular free wall and septum in HC group relative to controls. Further, QUE significantly decreases myocyte density relative to HC (Figures 5(a) and 5(b)). In contrast, in the left ventricle free wall, there were no differences between groups (Figure 5(c)).

### 3.4. Quercetin Prevented Cholesterol-Induced Decrease in Antioxidant Defenses

**Figure 6:**
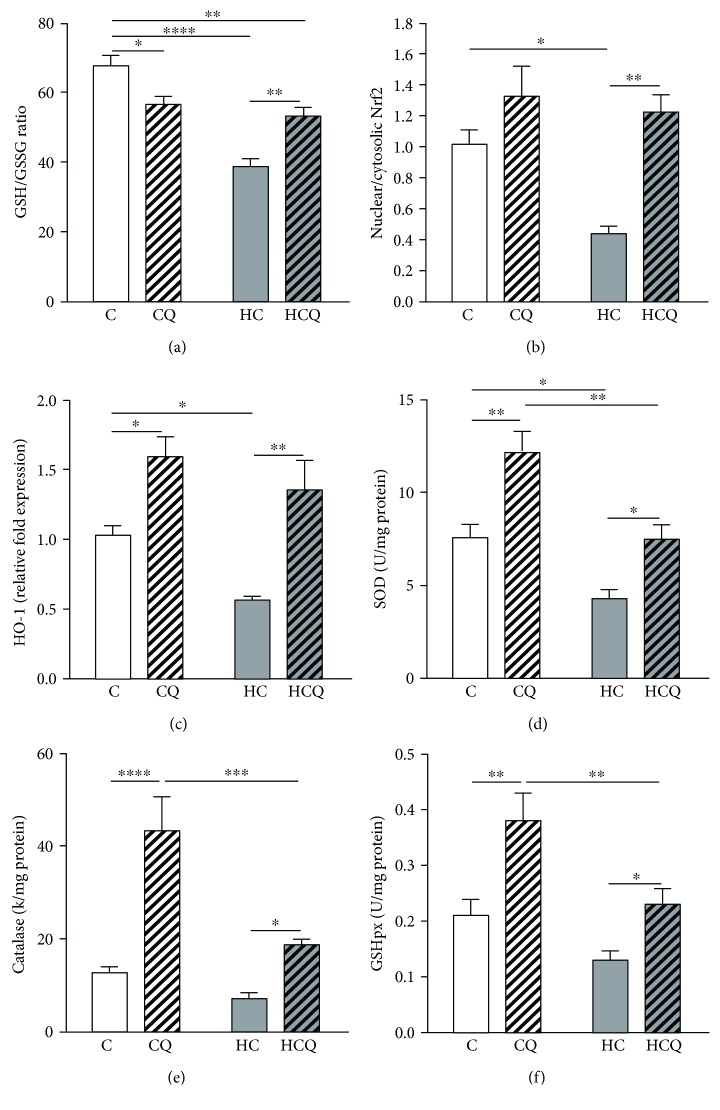
Quercetin protects against the decrease in cardiac antioxidant defenses induced by high-cholesterol diet. Antioxidant defenses (a) GSH/GSSG ratio, (b) nuclear translocation of Nrf2, (c) HO-1 expression, and activities of (d) SOD, (e) catalase, and (f) glutathione peroxidase in the heart from rats fed for 4 weeks with control diet (C), control diet containing 0.5% quercetin diet (CQ), high-cholesterol diet (HC), and high-cholesterol diet containing 0.5% quercetin (HCQ). Values are expressed as mean ± SEM. *N* = 6–8 rats/group. Two-way ANOVA test and Bonferroni posttest. Statistical differences: ^∗^ *p* < 0.05, ^∗∗^ *p* < 0.01, ^∗∗∗^ *p* < 0.001, and ^∗∗∗∗^ *p* < 0.0001.

The HC diet decreased the GSH/GSSG ratio by 43% (Figure 6(a)), Nrf2 translocation to the nucleus by 57% (Figure 6(b)), and HO-1 expression by 45% (Figures 6(b) and 6(c)). In addition, the HC decreased the activity of SOD by 43% (Figure 6(d)) and tended to decrease the activity of catalase (Figure 6(e)) and GSH peroxidase (Figure 6(f)). Quercetin ameliorated the decrease on HC-induced GSH/GSSG ratio by 47% (Figure 6(a)). Further, QUE increased the Nrf2 translocation to the nucleus by 30%, the HO-1 expression by 55% in control diet-fed rats (Figures 6(b) and 6(c)), and by 178% and 139%, respectively, in HC diet-fed rats (Figures 6(b) and 6(c)). Quercetin enhanced the activity of SOD (Figure 6(d)), catalase (Figure 6(e)), and GSH peroxidase (Figure 6(f)) by 61%, 240%, and 81% in control diet-fed rats, respectively (Figures 6(e) and 6(f)). When supplemented with HC diet, QUE enhanced the activity of SOD (Figure 6(d)), catalase (Figure 6(e)), and GSH peroxidase (Figure 6(f)) by 75%, 163%, and 77%, respectively (Figures 6(d), 6(e), and 6(f)). Conversely, ezetimibe prevented the decrease on GSH/GSSG ratio induced by HC diet (Supplementary Figure 2A).

### 3.5. Quercetin Reduced Cholesterol-Induced Oxidative Stress

**Figure 7:**
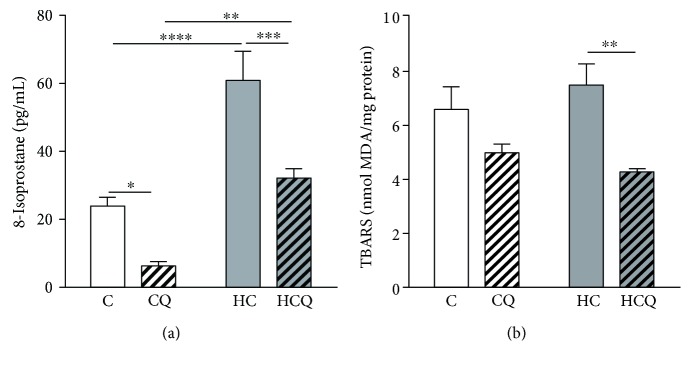
Quercetin protects against cardiac oxidative stress induced by high-cholesterol diet. (a) TBARS and (b) 8-isoprostane were measured in the heart from 12 h fasting rats fed for 4 weeks with control diet (C), control diet containing 0.5% quercetin diet (CQ), high-cholesterol diet (HC), and high-cholesterol diet containing 0.5% quercetin (HCQ). Values are expressed as mean ± SEM. *N* = 6–8 rats/group. Two-way ANOVA test and Bonferroni posttest. Statistical differences: ^∗^ *p* < 0.05, ^∗∗^ *p* < 0.01, ^∗∗∗^ *p* < 0.001, and ^∗∗∗∗^ *p* < 0.0001.

The HC diet increased the levels of 8-isoprostanes in the heart by 154% when compared to control group (Figure 7(a)). Further, QUE diminished 8-isoprostanes by 74% in control diet-fed rats and by 47% in HC diet-fed rats (Figure 7(a)). Although HC diet did not increase lipid peroxidation compared to control diet (Figure 7(b)), QUE reduced the lipid peroxidation in HC diet-fed rats by 43% (Figure 7(b)) and ezetimibe decrease it by 39% (Supplementary Figure 2B).

### 3.6. Quercetin Prevented Cholesterol-Induced Alteration in Bioenergetics

**Figure 8:**
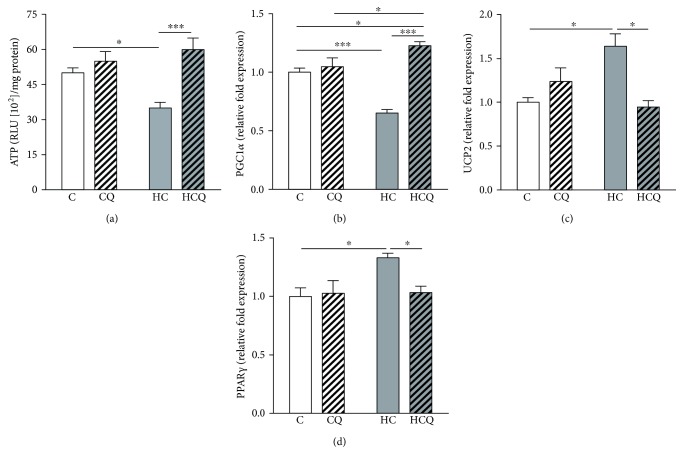
Quercetin protects against the decrease in ATP levels and the alterations of metabolic gene expression induced by high-cholesterol diet. (a) ATP levels, (b) *PGC-1α*, and (c) *UCP2* expression in the hearts of rats fed for 4 weeks with control diet (C), control diet containing 0.5% quercetin diet (CQ), high-cholesterol diet (HC), and high-cholesterol diet containing 0.5% quercetin (HCQ). Values are expressed as mean ± SEM. *N* = 6–8 rats/group. Two-way ANOVA test and Bonferroni posttest. Statistical differences: ^∗^ *p* < 0.05 and ^∗∗∗^ *p* < 0.001.

The HC diet decreased the cardiac levels of ATP by 30% (Figure 8(a)) and the expression of *PGC-1α* by 35% (Figure 8(b)) and increased the expression of *UCP2* by 64% and *PPARγ* by 33% (Figures 8(c) and 8(d)), relative to control diet. The supplementation with QUE prevented the decrease in ATP levels and *PGC-1α* expression (Figures 8(a) and 8(b)) and the increase in *UCP2* and *PPARγ* expression in the heart from HC diet-fed rats (Figures 8(c) and 8(d)). In contrast, QUE had no effect when supplemented to control diet. Conversely, ezetimibe prevented the decrease on ATP levels and *PGC-1α* expression and the increase on the expression of *UCP2* and *PPARγ* induced by HC diet (Supplementary Figures 3A–3D).

## 4. Discussion

In this study, we demonstrated that a HC diet induces oxidative stress, bioenergetic impairment, and cardiac dysfunction associated with structural alterations. Furthermore, we showed that a treatment with QUE was able to prevent most of the metabolic and cardiac alterations.

Considering that QUE prevents impairments in plasma glucose regulation in this HC diet model [12], we find it particularly interesting to elucidate cardiovascular dysfunction mechanisms under hyperglycemia and hypercholesterolemia conditions induced by a HC diet. Cardiovascular disease is the major cause of morbidity and mortality in subjects suffering from diabetes mellitus where coronary artery disease is the leading cause of cardiac complications [26]. Although the protective effect of QUE against cardiac dysfunction induced by high cholesterol has been reported [27–29], these effects have not been studied under hyperglycemic conditions. Still, neither the molecular nor the metabolic mechanisms have been addressed. The novelty of the present study lies in the clarification of the molecular mechanisms related to redox homeostasis and bioenergetics that cause morphological and functional impairments induced by cholesterol and the cardiac protection given by QUE.

### 4.1. Cholesterol Causes Cardiovascular Dysfunction

The detrimental effects on the cardiovascular system induced by a HC diet can be attributed to the increased cholesterol levels in plasma and by the alteration of the lipid profile: increased triglyceride, LDL, and VLDL levels and decreased HDL levels. It is noteworthy that the HC diet induced cholesterol accumulation in the heart, suggesting that the deleterious effects on cardiac function are beyond its known atherogenic effect [30]. However, there were no significant differences in the variables of systolic function or in the dynamic measurements of either ventricle in the HC group. Therefore, it is likely that these data support some initial degree of altered ventricular relaxation, which may be due to initial states of systemic metabolic and inflammatory instability, such as hypercholesterolemia [31, 32].

Oxidative stress and biogenetic impairment were totally prevented when the increase in total cholesterol plasma levels, and therefore the amount of cholesterol in the heart, caused by the HC diet was reversed by supplementation with ezetimibe, a drug used to treat hypercholesterolemia by blocking NPC1L1-dependent cholesterol transport [16]. Quercetin prevented the increase in total cholesterol in plasma induced by HC, but it only partially prevented the alterations in lipid profile levels, as similarly reported in hypercholesterolemic Apo E^−/−^ mice [27]. Thus, the partial effect of QUE on preventing the HC diet-induced increase in LDL and VLDL levels supports the idea that the protective effect of QUE on cardiovascular function is beyond its effect on controlling total cholesterol levels.

### 4.2. Quercetin Protects against Oxidative Stress Induced by Cholesterol

Oxidative stress has a major role in the development of diabetic cardiomyopathy [33]. Therefore, we studied the prooxidant effects associated with the direct incorporation of cholesterol into the myocardium and cardiac dysfunction under hyperglycemia [12]. We found a prooxidant state in cardiac tissue, characterized by a decrease in antioxidant defenses and increased lipid peroxidation in HC diet-fed rats compared with control rats. Accumulation of cholesterol in the pancreas, leading to elevated oxidative stress, induced *β*-cell dysfunction with an impaired glucose-stimulated insulin secretion [12, 34]. Here, we consistently found increased cholesterol levels in cardiac tissue associated with oxidative stress and diastolic dysfunction in HC diet-fed rats.

Diastolic dysfunction, a recognized feature of diabetic cardiomyopathy [35] and alteration of ventricular relaxation, is associated with an increased ROS production from myocardium and endothelial sources [36]. Increased levels of cardiac ROS may explain some of the changes in Ca^2+^ handling proteins and the increased Ca^2+^ sensitivity of myofilaments that prompt the development of diastolic dysfunction and some types of arrhythmias [37]. Increased mitochondrial ROS have been observed in animal models of pressure overload and ventricular dysfunction [38] and in prediabetic animals with mild diastolic dysfunction [39]. Progression of diastolic dysfunction is associated with mitochondrial oxidative stress in a diet-induced diabetes model [40]. All of the above may induce systemic hypertension and cardiac remodeling. Although in our study we did not measure *in vivo* systemic arterial pressure, our finding suggests that the HC animals might be hypertensive and that QUE reverts this cardiovascular phenotype. In fact, the left ventricle wall of the HC animals was thicker and with a reduced lumen, a classical phenotype of cardiac remodeling due to an increased afterload, and these effects were prevented by QUE. Therefore, the use of antioxidants that attenuates mitochondrial injury may improve myocardial response to hemodynamic overload and optimize relaxation, decreasing left ventricular diastolic dysfunction. Interestingly, the mitochondria-targeted antioxidant mitoTEMPO prevented diastolic dysfunction in diabetic mice [41]. We found consistently that QUE, a polyphenolic antioxidant that accumulates in mitochondria [42, 43], protected against HC diet-induced diastolic dysfunction.

The role of Nrf2 has been widely described as a transcription factor related with cardioprotection [44]. The major aspects of Nrf2 are dependent on Nrf2-induced genes and their proteins including heme oxygenase-1 (HO-1). HO-1 by-products, such as carbon monoxide biliverdin and bilirrubin, exert beneficial effects through the protection against oxidative damage, vascular inflammation, and cell death [45]. Even more, several studies have shown the cardiovascular benefits of HO-1 as reviewed elsewhere [46].

Quercetin has been shown to suppress LPS-induced oxidant production and adhesion molecule expression, crucial in initiation and progression of atherosclerosis, through the induction of Nrf2 activation and antioxidant enzyme expression in human aortic endothelial cells [47]. In the heart, QUE attenuated changes in cardiac function induced by high-carbohydrate/high-fat diet and prevented the decrease of Nrf2 and HO-1 expressions [48]. Here, we report for the first time the cardioprotective effect of QUE through Nrf2 mechanisms, against the damage induced by a HC diet.

A wide range of evidence supports the cardioprotective effects of QUE relying mainly on its antioxidant properties [49, 50]. The antioxidant properties of QUE have been attributed to its ability to protect mitochondria and induce biogenesis by activating the Nrf2 signaling pathway [51]. Although the role of QUE in bioenergetics has been studied in cardioprotection in an ischemia-reperfusion model [52], this has not been addressed under a hypercholesterolemic/hyperglycemic model.

### 4.3. Quercetin Protects against the Alteration in Bioenergetics Induced by Cholesterol

Considering that ATP consumption per tissue weight in the heart is the highest in the body and that this organ has limited energy reserves, cardiac bioenergetics and mitochondrial function are a highly regulated and vital process [53, 54]. Adequate bioenergetics leading to maintaining ATP levels is essential to preserve the physiological function of the cell and tissues. In fact, we have reported that the HC diet promotes insulin release impairment, which causes hyperglycemia, by inducing bioenergetic deficiency in insulinoma cells [12]. In this study, we found that this HC diet also induces a reduction in ATP levels in the heart, causing diastolic and morphological impairments, which were reversed by QUE treatment. From this viewpoint, a constant flux of ATP is essential to maintaining the cardiac contractile function [55]. Interestingly, it has been suggested that alterations in cardiac energy metabolism play a key role in the pathogenesis of diabetic cardiomyopathy [56]. Heart failure is associated with decreased mitochondrial biogenesis and function in both the heart and skeletal muscles, supporting the concept of a systemic mitochondrial cytopathy [57], which may be triggered by a HC diet.

Cardiac intermediary metabolism and its transcriptional networks are of interest to understanding the regulation of cardiac energetics, and PGC-1*α* plays a central role in these pathways [55]. PGC-1*α* is a key regulator of mitochondrial biogenesis, by inducing several genes involved in the tricarboxylic acid (TCA) cycle and antioxidant defense. In addition, PGC-1*α* induces the mitochondrial transcription factor A (Tfam), an important factor for mitochondrial DNA (mtDNA) transcription, translation, and repair [58]. In this study, we found that a HC diet decreased *PGC-1α* and increased *UCP2* expression. PGC-1*α* and mitochondrial function restoration have been associated with cardiac function improvement [59]. We found that QUE prevented the HC diet-induced decrease in PGC-1*α* and attenuated diastolic dysfunction. Lipid accumulation in the myocardium has detrimental effects on PGC-1*α* expression and mitochondrial function, thus promoting contractile dysfunction [59]. In our study, a HC diet promoted cholesterol accumulation in cardiac tissue that was associated with a reduction in mRNA PGC-1*α* levels. The protein UCP2 is located in the mitochondrial inner membrane where it could act as a regulated protonophore, dissipating the mitochondrial membrane potential and thus decreasing the ATP production linked to oxygen consumption. In this regard, the HC diet may also impair intracellular bioenergetics by promoting *UCP2* expression, as was found in this study. UCP2 (mRNA and protein expression) caused decreased cardiac mechanical efficiency (hydraulic work/myocardial oxygen consumption) in a septic shock model in rats [60] and was associated with an impaired myocardial energy metabolism and decreased ATP levels in a canine endotoxin shock model [61]. Increased UCP2 has been correlated with reduced cardiac efficiency in cardiac hypertrophy in hyperthyroidism [62]. It is noteworthy that mitochondrial uncoupling occurs in the hearts of diabetic db/db mice, mainly mediated by increased UCP activity, causing a deterioration of contractile function [63].

Although PPAR*γ* is not as highly expressed in cardiac tissue as PPAR*α*, it is still critical for cardiac function [64]. Remarkably, human hearts contain 8- to 15-fold higher PPAR*γ* mRNA levels than mouse hearts [65], suggesting that PPAR*γ* is more functional in humans, although its detrimental or beneficial effect on myocardial function is still controversial [66]. In the present study, the deleterious effect of a HC diet on cardiac function was accompanied by an increase on PPAR*γ* expression. It has been shown that overexpression of PPAR*γ* in mice created a dilated cardiomyopathy associated with increased lipid stores and altered mitochondrial architecture [65]. These effects were exacerbated under hyperglycemic conditions in a streptozotocin-induced diabetes model with increased cardiac PPAR*γ* expression in mice [65].

The protective effect of QUE against HC diet-induced diastolic dysfunction may lie in its ability to prevent cholesterol accumulation and an ATP reduction and to preserve PGC-1*α* and UCP2 expression. It has been suggested that at least part of the cardioprotective effects of QUE resides in its ability to protect against mitochondrial damage in an ischemia/reperfusion model in rats [52]. Quercetin has been shown to prevent the increase of UCP2 expression in the liver from high-sucrose and HC diet-fed rabbits, which was associated with a decrease in lipid peroxidation and an increase in GSH content in this organ [67].

In conclusion, the molecular mechanisms that support the cardioprotective effects of QUE supplementation in rats exposed to a HC diet may be mediated by the upregulation of intracellular antioxidant mechanisms and improved bioenergetics of the heart. From this point of view, the mitochondria can be considered a pharmacological target to modulate structural and functional changes of the heart under impaired metabolic conditions. Exposure to high-cholesterol diets and the occurrence of oxidative stress and mitochondrial dysfunction are clearly important variables to consider in the follow-up of patients with increased cardiovascular risk.

## Figures and Tables

**Table 1:** Cardiac structural and functional parameters determined by *in vivo* echocardiography.

	C	CQ	HC	HCQ
Cardiac parameters				
Ejection fraction (%)	75.2 ± 7.1	72.5 ± 6.3	69.1 ± 6.7	72.7 ± 7.3
Fractional shortening (%)	42.3 ± 3.5	45.7 ± 5.1	40.8 ± 2.3	41.5 ± 4.8
Heart rate, bpm	215 ± 7.1	221 ± 19	231 ± 23	218 ± 17
AWT, mm	1.6 ± 0.3	1.9 ± 0.4	2.3 ± 0.4	2.1 ± 0.4
LVEDC, mm	5.2 ± 1.7	5.4 ± 1.5	4.9 ± 1.1	4.8 ± 0.5
LVESC, mm	2.3 ± 0.8	2.1 ± 0.9	2.4 ± 0.6	2.2 ± 0.7
RVEDC, mm	1.9 ± 0.5	2.1 ± 0.7	2.2 ± 0.6	1.8 ± 0.4
RVESC, mm	2.7 ± 0.6	2.4 ± 0.5	3.0 ± 0.8	2.9 ± 0.4
Aortic				
Vmax, m/s	2.1 ± 0.9	1.8 ± 0.7	1.7 ± 0.5	2.0 ± 0.8
Vmean, m/s	1.1 ± 0.4	0.9 ± 0.5	0.9 ± 0.3	1.2 ± 0.4
Peak G max, mmHg	21.4 ± 6.2	18.3 ± 5.2	16.8 ± 4.1	19.5 ± 4.2
Peal G mean, mmHg	5.5 ± 1.7	4.3 ± 1.4	4.1 ± 0.9	5.2 ± 0.4
IVRT, ms	22.6 ± 1.5	25.1 ± 2.7	24.9 ± 3.9	26.7 ± 2.9

Values are mean ± SEM. AWT: aortic wall thickness; LVEDC: left ventricular end-diastolic cavity; LVESC: left ventricular end-systolic cavity; RVEDC: right ventricular end-diastolic cavity; RVESC: right ventricular end-systolic cavity; Vmax: maximal velocity; G: gradient; IVRT: isovolumetric relaxation time.

## Supplementary Materials

Supplementary Materials Supplementary Figure 1: ezetimibe protects againts the increase on total cholesterol in plasma, cholesterol content in heart and heart weight induced by a high cholesterol diet. In rats fed for 4 weeks with control, diet (C), high cholesterol diet (HC) or high cholesterol diet containing 0.001% ezetimibe (HCE) the A) total cholesterol in plasma, B) cholesterol content in heart and C) the weight of the heart were measured after 12 h, fasting. Values are expressed as mean ± SEM. *N* = 6-8 rats/group. All one-way ANOVAs, Bonferroni post-test significances showed in each figure ^∗^ *p* < 0.05, ^∗∗^ *p* < 0.01 and ^∗∗∗∗^ *p* < 0.0001. Supplementary Figure 2: ezetimibe protects againts the alteration of the cardiac oxidative status induced by a high cholesterol diet. In heart from rats fed for 4 weeks with control diet (C), high cholesterol diet (HC) or high cholesterol diet containing 0.001% ezetimibe (HCE) the A) the GSH/GSSH and B) Lipid peroxidation were measured after 12 h fasting. Values are expressed as mean ± SEM. *N* = 6–8 rats/group. All one-way ANOVAs, Bonferroni post-test significances showed in each figure. ^∗∗∗^ *p* < 0.001 and ^∗∗∗∗^ *p* < 0.0001. Supplementary Figure 3: ezetimibe protects againts the cardiac metabolic alterations induced by a high cholesterol diet. In heart from rats fed for 4 weeks with control diet (C), high cholesterol diet (HC) or cholesterol diet containing 0.001% ezetimibe (HCE) the A) ATP levels and the expression of B) PGC-1*α* C) UCP2 and D) PPAR*γ* were measured after 12 h fasting. Values are expressed as mean ± SEM. *N* = 6-8 rats/group. All one-way ANOVAs, Bonferroni post-test significances showed in each figure. ^∗∗^ *p* < 0.01, ^∗∗∗^ *p* < 0.001 and ^∗∗∗∗^ *p* < 0.0001. Supplementary Table 1: list of primers used in this study for RT-PCR.ACTB, Beta-actin; GAPDH, Glyceraldehyde-3-phosphate dehydrogenase; PGC-1*α*, Peroxisome proliferator-activated receptor gamma coactivator 1 alpha; PPAR*γ*, Peroxisome proliferator-activated receptor gamma; UCP2, mitochondrial uncoupling protein 2.
