# Supplementary material for: Quercetin Prevents Diastolic Dysfunction Induced by a High-Cholesterol Diet: Role of Oxidative Stress and Bioenergetics in Hyperglycemic Rats
Source: Oxid Med Cell Longev. 2018 Jan 11;2018:7239123. doi: 10.1155/2018/7239123 (PMC5821945; doi:10.1155/2018/7239123)
Supplement: Supplementary Materials — Supplementary Figure 1: ezetimibe protects againts the increase on total cholesterol in plasma, cholesterol content in heart and heart weight induced by a high cholesterol diet. In rats fed for 4 weeks with control, diet (C), high cholesterol diet (HC) or high cholesterol diet containing 0.001% ezetimibe (HCE) the A) total cholesterol in plasma, B) cholesterol content in heart and C) the weight of the heart were measured after 12 h, fasting. Values are expressed as mean ± SEM. N = 6-8 rats/group. All one-way ANOVAs, Bonferroni post-test significances showed in each figure ∗ p < 0.05, ∗∗ p < 0.01 and ∗∗∗∗ p < 0.0001. Supplementary Figure 2: ezetimibe protects againts the alteration of the cardiac oxidative status induced by a high cholesterol diet. In heart from rats fed for 4 weeks with control diet (C), high cholesterol diet (HC) or high cholesterol diet containing 0.001% ezetimibe (HCE) the A) the GSH/GSSH and B) Lipid peroxidation were measured after 12 h fasting. Values are expressed as mean ± SEM. N = 6–8 rats/group. All one-way ANOVAs, Bonferroni post-test significances showed in each figure. ∗∗∗ p < 0.001 and ∗∗∗∗ p < 0.0001. Supplementary Figure 3: ezetimibe protects againts the cardiac metabolic alterations induced by a high cholesterol diet. In heart from rats fed for 4 weeks with control diet (C), high cholesterol diet (HC) or cholesterol diet containing 0.001% ezetimibe (HCE) the A) ATP levels and the expression of B) PGC-1α C) UCP2 and D) PPARγ were measured after 12 h fasting. Values are expressed as mean ± SEM. N = 6-8 rats/group. All one-way ANOVAs, Bonferroni post-test significances showed in each figure. ∗∗ p < 0.01, ∗∗∗ p < 0.001 and ∗∗∗∗ p < 0.0001. Supplementary Table 1: list of primers used in this study for RT-PCR.ACTB, Beta-actin; GAPDH, Glyceraldehyde-3-phosphate dehydrogenase; PGC-1α, Peroxisome proliferator-activated receptor gamma coactivator 1 alpha; PPARγ, Peroxisome proliferator-activated receptor gamma; UCP2, mitochondrial uncoupling [file 7239123.f1.docx]

**Supplementary Table 1:** List of primers used in this study for RT-PCR.

| **Gene** | **Forward** | **Reverse** |
| --- | --- | --- |
| ***PPARγ*** | GGAGTCCATGCTTGTGAAGGA | CCCAAACCTGATGGCATTGTG |
| ***PGC-1α*** | TGGAGTGACATAGAGTGTGCTG | TATGTTCGCGGGCTCATTGT |
| ***UCP2*** | CGAGCCTTCTACAAGGGGTTC | AAAGACAGGGCAGGAATGGG |
| ***GAPDH*** | TCTCTGCTCCTCCCTGTTCT | TACGGCCAAATCCGTTCACA |
| ***ACTB*** | AGATCAAGATCATGGCTCCTCCT | AGGGTGTAAAACGCAGCTCA |

ACTB, Beta-actin; GAPDH, Glyceraldehyde-3-phosphate dehydrogenase; PGC-1α, Peroxisome proliferator-activated receptor gamma coactivator 1 alpha; PPARγ, Peroxisome proliferator-activated receptor gamma; UCP2, mitochondrial uncoupling protein 2.
